# Automated intracorporeal tool exchange with the Symphera multi-tool prototype: a crossover evaluation of efficiency and surgeon workload in a porcine laparoscopic model

**DOI:** 10.1007/s00464-026-13190-w

**Published:** 2026-09-21

**Authors:** Fabian Haak, Conrad Heid, Brett Colton Parker, Antonello Forgione, Jacques Marescaux

**Affiliations:** 1 Division of Hepatobiliary Surgery and Visceral Surgery, Department of Visceral, Transplant, Thoracic and Vascular Surgery, University Hospital Leipzig, Liebigstraße 24, 04103 Leipzig, Germany; 2 Symphera GmbH, Munich, Germany; 3 Division of Minimally Invasive Surgery, Department of Surgery, The Johns Hopkins University School of Medicine, Baltimore, MD USA; 4 IRCAD (Institut de Recherche Contre Les Cancers de L´Appareil Digestif), Louis Pasteur University, Strasbourg, France

**Keywords:** Minimally invasive surgery, Laparoscopic instruments, Instrument exchange, Surgical workflow, Intracorporeal tool switching, Workload

## Abstract

**Background:**

Conventional laparoscopic instrument exchange requires repeated trocar withdrawal, redirecting surgeon gaze and disrupting operative flow. The Symphera system enables intracorporeal tool-tip switching, but its impact on procedural efficiency, visual attention, and workload has not been evaluated in a controlled setting.

**Methods:**

Two surgical fellows performed three standardised laparoscopic tasks (cholecystectomy, controlled hepatic bleeding, dissection–coagulation relay) under Symphera and conventional conditions in a live porcine crossover model. Primary outcomes were total task time and tool change time; secondary outcomes included gaze behaviour (eyes-on proportion, disruption events), subjective workload (SURG-TLX), device usability, and heart rate.

**Results:**

Mean total task time was 27.3 ± 10.9 vs. 33.9 ± 13.2 min (−19.6%; HL estimate −6.7 min, 95% CI −12.4 to −1.5; *p* = 0.10). Tool change time was reduced by 61.5% (3.6 ± 2.3 vs. 9.4 ± 6.6 min; HL estimate −5.2 min, 95% CI −9.7 to −3.4; *p* = 0.10). Intracorporeal exchanges were completed in a mean of 4.3 ± 4.4 s (75% within 5 s) versus 25.3 ± 23.4 s for extracorporeal exchanges. Eyes-on proportion was higher with Symphera (86.0 ± 10.9% vs. 78.1 ± 9.8%; HL estimate + 7.7 pp, 95% CI + 0.9 to + 14.8; *p* = 0.031), with instrument exchange as a gaze disruption cause markedly reduced. SURG-TLX scores were 44.8% lower with Symphera (40.2 ± 15.8 vs. 72.9 ± 19.8), with reductions across all six dimensions.

**Conclusion:**

Symphera demonstrated favourable trends in procedural efficiency, gaze behaviour, and subjective workload versus conventional instrument exchange. The convergence of behavioural, subjective, and efficiency data supports the hypothesis that intracorporeal exchange preserves visual attention and may reduce perceived workload. Larger, adequately powered studies are warranted.

**Supplementary Information:**

The online version contains supplementary material available at https://doi.org/10.1007/s00464-026-13190-w.

Minimally invasive surgery (MIS) has transformed surgical practice over recent decades, reducing post-operative morbidity, shortening hospital stays, and improving patient recovery [1–4]. Despite these advantages, MIS remains burdened by persistent workflow inefficiencies, most notably the repeated extracorporeal instrument exchanges that characterise conventional laparoscopic technique [5–8]. Each conventional instrument exchange requires the surgeon to withdraw the instrument from the trocar and oftentimes redirect their gaze away from the operative monitor to manually or verbally select the replacement instrument, then re-engage the operative field—a sequence that may occur repetitively throughout a single procedure. Cumulatively, these interruptions contribute to prolonged operative time, increased cognitive load, and reduced visual attention to the surgical field, with downstream implications for surgeon fatigue, procedural safety, and institutional cost [9–14]. Several strategies have been introduced to reduce this burden. Multifunctional instruments consolidating multiple capabilities into a single device have shown operative time reductions of 17–25% in experimental settings, and automated intracorporeal exchange prototypes—which eliminate the need to withdraw the instrument shaft entirely—have demonstrated further workflow improvements, though none have reached clinical adoption [15–18].

The Symphera system addresses this problem by enabling intracorporeal tool-tip switching—allowing surgeons to exchange select instruments without withdrawing the device from the trocar or leaving the operative field. In an earlier formative evaluation of the fourth-generation prototype, seven experienced surgeons completed standardised tasks using excised animal tissue [19]. The study demonstrated that the automated exchange sequence could be completed in a median of 3.4 s, with no safety concerns. Questionnaire responses indicated strong enthusiasm for the device’s potential to streamline operative workflows, though ergonomic refinements and software stability were identified as priorities for further development.

That evaluation, whilst informative, was limited by its formative design: it included no control comparator, used excised tissue rather than a live surgical model, and did not assess the downstream effects of faster instrument exchange on visual attention or perceived workload. Demonstrating that intracorporeal exchange translates into meaningful gains in these dimensions—rather than simply measuring the speed of the exchange event itself—requires a controlled comparative study in a more realistic operative setting.

The present study addresses this gap. We report a prospective, within-subject crossover evaluation comparing the Symphera prototype against conventional extracorporeal instrument exchange across three standardised laparoscopic tasks performed in a live porcine model at IRCAD France. Our primary aim was to assess whether intracorporeal exchange reduces total task time and tool change time relative to conventional laparoscopic technique. Secondary aims were to characterise the effect on surgeon gaze behaviour, subjective workload, device usability, and physiological stress. Together, these outcomes were selected to test a working hypothesis: that replacing extracorporeal with intracorporeal exchange preserves surgeon visual attention on the operative field, which may in turn reduce the accumulation of cognitive load across an operative case. This study was not designed or powered to evaluate safety outcomes.

## Methods

### Study design

This was a prospective, single-centre, within-subject crossover study with counterbalanced condition order (non-randomised) evaluating the Symphera prototype (Ver 5.0) against a standard set of conventional laparoscopic instruments during standardised laparoscopic procedures in a live porcine model. This manuscript was prepared in accordance with the STROBE reporting guidelines for observational studies; the completed checklist is provided as Supplementary File 1. The study employed a within-subject crossover design in which each participant performed a standardised set of surgical tasks under both conditions. Conditions were counterbalanced across participants and days: on Day 1, Participant 1A began with Symphera and Participant 2B with Control; on Day 2, the order was reversed. The study was conducted at IRCAD France, Strasbourg between 11 and 12 September 2025. The study was approved by ICOMETH (registration number: 38.2025.02.015; approval date: 5 September 2025) and all participants provided written informed consent.

### Participants

Two surgical fellows from IRCAD (referred to as Participant 1A and Participant 2B) participated in the study; both were male, reflecting the composition of the available fellow pool at the time of recruitment, with no sex-based exclusion criteria applied. Both participants had completed their core laparoscopic training (Professional experience in laparoscopy in years: from 6 to 15) and were experienced in standard laparoscopic instrument handling (Participant age: 35 and 42). Neither participant had prior exposure to the Symphera system before the study. All participants provided written informed consent prior to enrolment. The sample size reflects the exploratory, within-subject design of this evaluation: with each participant completing three tasks under each condition on two separate operating days, the study was structured to generate detailed, repeated-measures performance data rather than to achieve population-level statistical power.

## Experimental conditions

### Symphera condition

In the Symphera condition, participants used the Symphera prototype (Ver 5.0), which enables intracorporeal instrument changes without withdrawing the instrument shaft from the trocar. To perform an exchange, the active tool tip is retracted proximally into the device body, after which a rotating barrel is turned to select the desired tool tip from up to seven available slots. Once selected, the new tool tip is automatically reintroduced into the distal end of the instrument and advanced into the operative field. Barrel positions can be loaded individually, allowing a customised instrument configuration for each procedure. In this study, five tool tips were loaded across all procedures: scissors, monopolar hook, bipolar forceps, grasper, and Overholt dissector. The device is compatible with an 8 mm trocar. A demonstration of the automated intracorporeal tool-tip exchange sequence is provided in Supplementary Video 1. Extracorporeal exchanges were still required in the Symphera condition for instruments outside the platform’s scope—including the clip applier, suction/irrigation device—and for cleaning of tool tips when necessary. These instrument categories were used identically in both conditions and recorded as extracorporeal exchanges in all cases.

### Control condition

In the Control condition, participants used a standard set of conventional laparoscopic instruments (monopolar hook, bipolar forceps, Overholt dissector, scissors, and grasper) with standard extracorporeal instrument exchange. Each tool change required full withdrawal of the instrument from the trocar, manual exchange at the instrument table, and re-insertion through the trocar. No advanced energy devices were used in either condition.

### Surgical tasks

Each participant performed three standardised surgical tasks under each condition on each study day, yielding a total of 12 tasks per participant (six Symphera, six Control) and 24 tasks across the study. Each set of three tasks was performed sequentially within a single porcine model, with a dedicated animal used for each condition per participant per day, giving a total of eight animals across the study. The tasks were as follows:

*Task 1–Cholecystectomy* A routine laparoscopic cholecystectomy was performed in the porcine model, comprising dissection and display of Calot’s triangle, clipping and division of the cystic duct and cystic artery, removal of the gallbladder from the liver bed, and haemostasis of the liver bed where required.

*Task 2–Controlled Hepatic Bleeding* A standardised bleeding control task was performed on the porcine liver. A 3 cm capsular incision was made using laparoscopic scissors, and the participant then switched to bipolar forceps to achieve haemostasis. This sequence was repeated 10 times, providing a controlled and reproducible measure of instrument exchange frequency and haemostatic performance.

*Task 3–Dissection-Coagulation Relay* A standardised dissection and coagulation task was performed requiring sequential instrument exchanges across two anatomical sites. Each cycle comprised four steps: (1) division of a vessel on the gastroepiploic arcade using scissors, (2) intentional injury to a vessel on the mesentery of the small intestine using a monopolar hook, (3) coagulation of the gastroepiploic arcade vessel using bipolar forceps, and (4) coagulation of the small intestinal mesentery vessel using a grasper and monopolar hook.

Tasks were performed in a fixed order (Task 1–Cholecystectomy, Task 2–Controlled Hepatic Bleeding, Task 3–Dissection-Coagulation Relay) across all participants, conditions, and days. The use of a fixed task order was intended to standardise the procedural sequence across all sessions; however, it does not preclude sequence effects or within-day fatigue and familiarity confounds, which should be considered when interpreting task-level comparisons. Procedures were performed in a porcine model under standard laparoscopic conditions, with carbon dioxide insufflation. A standardised four-port configuration was used across all tasks and in both conditions: a 12 mm camera trocar, a 12 mm trocar for the primary working instrument (Symphera device or the corresponding conventional instrument), a 5 mm trocar for the surgeon’s second working hand, and a 5 mm assistant trocar used for retraction. Although the Symphera device is compatible with an 8 mm trocar, a 12 mm trocar was used because no smaller trocars were available at the study site. The same port setup was maintained throughout to avoid introducing a confounding variable between conditions. The laparoscopic camera was held in a fixed position using a mechanical retractor. A surgical assistant was present to support with camera positioning, retraction using a grasper through the assistant trocar during Cholecystectomy, or suction and irrigation if requested by the surgeon.

## Outcome measures

### Procedural efficiency

Total task time was defined as the time from a verbal command to start until completion of all described procedural steps, and was recorded in minutes. The number of tool changes and total tool change time were recorded for each procedure by analyzing video recordings of each task. A tool change was defined as any event in which one instrument was replaced by another at the operative site. Tool changes were classified as either intracorporeal (Symphera condition only; exchange performed within the trocar without instrument withdrawal) or extracorporeal (instrument fully withdrawn and exchanged outside the body). Individual tool change durations were recorded in seconds. For intracorporeal changes, duration was measured from initiation of the retraction mechanism—defined as the moment the active tool tip began retracting into the device body—to reintroduction of the newly selected tool tip at the distal end of the instrument. For extracorporeal changes, duration was measured from the moment the instrument began to be withdrawn from the trocar to the moment the new instrument had been fully reinserted through the trocar and returned to the operative focus point. Mean change time per exchange was calculated as total tool change time divided by the number of tool changes.

### Gaze analysis

Surgeon gaze direction was recorded throughout each procedure using manual video annotation by an independent reviewer. Two camera angles were used simultaneously: a full overview angle capturing the operative field and the surgeon’s body, and a head-on angle providing a full-face view to enable reliable gaze classification. Gaze state was classified at each time point into one of three categories following the framework established by Ibbotson et al.: Eyes on (gaze directed at the laparoscopic monitor), Eyes down (gaze directed downward towards the hands and/or instrument exchange area), or Eyes off (gaze directed away from both monitor and hands) [20]. Eyes-on proportion was calculated as the percentage of total task time during which the surgeon’s gaze was classified as Eyes on.

Disruption events—defined as transitions from the Eyes on gaze state to any other gaze state—were recorded and classified by reason following the taxonomy described by Sutton et al.[21] Four disruption categories were used: instrument exchange, extracorporeal work, equipment troubleshooting, and communication. The relative frequency of each disruption reason was expressed as a percentage of total disruption events per participant and condition.

### Subjective workload

Subjective workload was assessed using the Surgery Task Load Index (SURG-TLX), a validated six-dimension instrument adapted from the NASA-TLX for surgical settings [22]. The six dimensions are: Mental Demands, Physical Demands, Temporal Demands, Task Complexity, Situational Stress, and Distractions. The SURG-TLX consists of two parts. First, dimension weights are derived from 15 pairwise comparisons, with each dimension receiving a score between 0 and 5 reflecting its relative contribution to perceived workload. Second, each dimension is rated on a 20-point bipolar Likert scale anchored between low and high endpoints specific to each dimension. The weighted total score (range 0–100) was calculated as the sum of weighted dimension scores. The SURG-TLX was administered once per condition, immediately after completion of all three tasks on each operating day.

### Device usability questionnaire

Following each operating day, participants completed a post-operative device usability questionnaire. The questionnaire comprised 22 items grouped into six domains: Ergonomics, Haptics, Endurance, Workflow, Controls, and Overall assessment. Each item was rated on a 5-point Likert scale. For items where a lower raw score indicated a more favourable response, scores were direction-corrected so that higher values consistently indicated more favourable ratings (range: 1–5). Domain scores were computed as the mean of constituent item scores.

### Physiological stress

Surgeon heart rate (HR) was recorded continuously throughout each procedure using two wrist-based Polar Vantage M watches (Device-ID: 59256A25 and 58FDC22D). As wrist-based optical monitors, these devices provide a practical but relatively low-resolution proxy for physiological stress compared with chest-strap or ECG-based systems. Mean heart rate (bpm) was calculated over the total task duration for each procedure. Within-task heart rate trajectories were derived by normalising task duration to a common time axis (0–100%) and computing mean HR at each normalised time point across procedures within each condition and participant.

### Data collection and processing

All procedures were recorded on video using Akaso EK 7000 cameras for close-up videos, and both a Samsung Galaxy S20FE and a Fairphone 5 for full OR coverage. Task times, tool change events, and gaze states were annotated from video review by the first author (F.H.) and the second author (C.H.). Individual tool change durations were measured by stopwatch during video review. Raters were not blinded to condition, as the device in use (Symphera or conventional instruments) was visually apparent in all recordings. Prior to independent annotation, both raters jointly reviewed one complete porcine case—comprising all three tasks—to align their interpretation of the start and end points of each event type across all task formats. Annotation of the remaining recordings was shared equally between the two raters. Large language model (LLM)-based artificial intelligence tools (Claude, Anthropic) were used to assist with language editing. All scientific content, data, analyses, and conclusions are the sole responsibility of the authors.

### Statistical analysis

Descriptive statistics are reported as mean ± standard deviation (SD) and median where appropriate. Given the small sample size (*n* = 2 participants, 3 procedures per condition per participant), non-parametric methods were used for inferential testing. The Wilcoxon signed-rank test was used to compare paired observations between the Symphera and Control conditions for continuous outcomes (total task time, tool change time, and heart rate). For eyes-on proportion, a pooled Wilcoxon signed-rank test was applied across all paired observations from both participants; participant-level results are additionally reported in the Results section. For the comparison of extracorporeal change durations between conditions, a Wilcoxon rank-sum test was applied to the pooled set of individual change events; because these 614 events are clustered within procedures and participants, the independence assumption is not fully met and this p-value should be interpreted with caution. A two-sided significance level of *α* = 0.05 was used throughout. Where applicable, 95% confidence intervals were estimated as the Hodges–Lehmann location shift (paired tests) or pseudo-median difference (unpaired test) using the standard inversion of the Wilcoxon test statistic. For outcomes where only two paired observations were available per participant, confidence intervals could not be constructed and only point estimates are reported.Given the exploratory nature of the study, no correction for multiple comparisons was applied. No missing data were encountered; all recorded tasks were complete and included in the analysis.

Analyses were performed using R version 4.4.2 (R Core Team, 2024; R Foundation for Statistical Computing, Vienna, Austria).

## Results

**Fig. 1:**
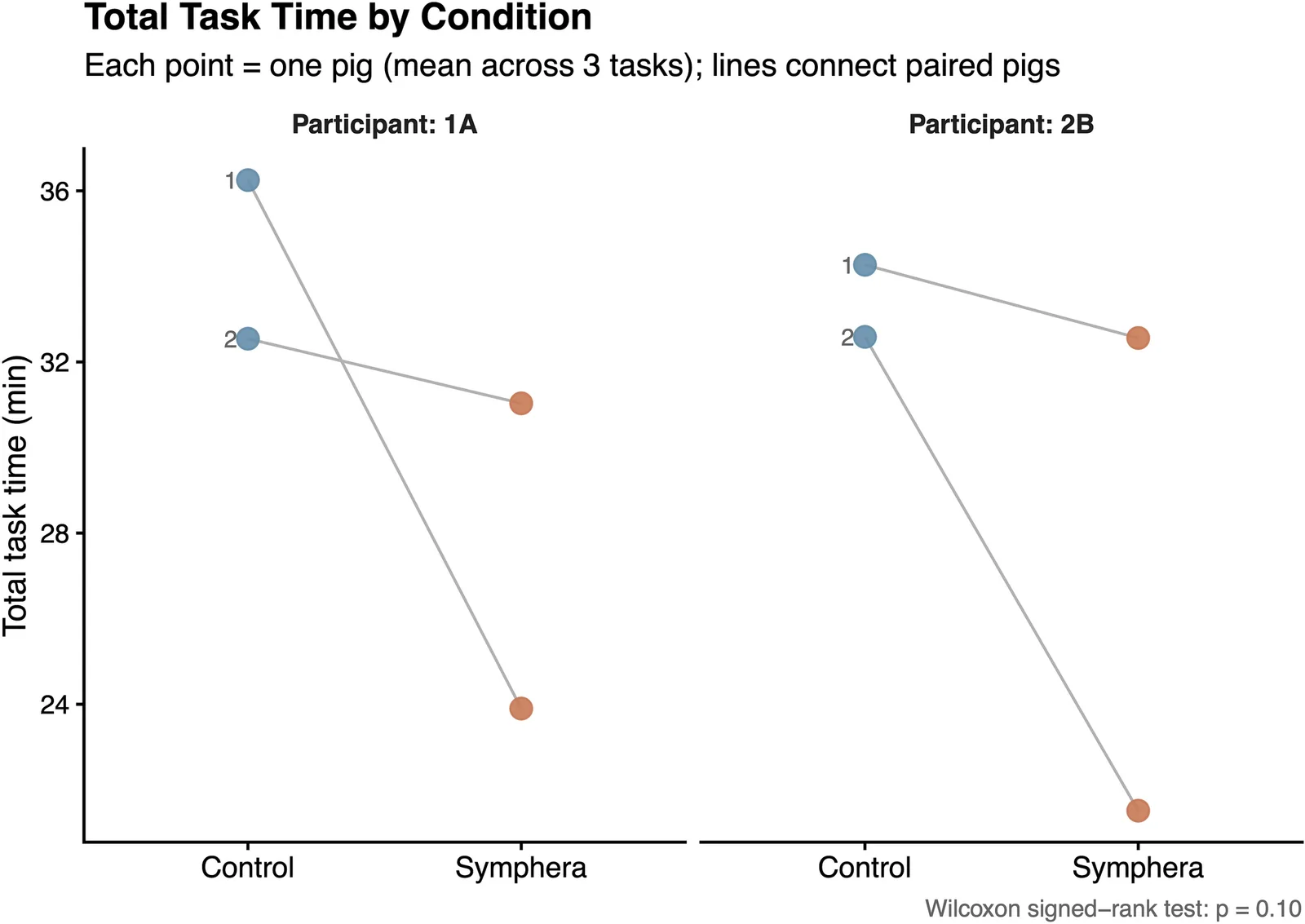
Total task time (minutes) by condition and participant. Box plots show mean ± standard deviation for the Symphera and Control conditions across all three standardised laparoscopic tasks (Cholecystectomy, Controlled Hepatic Bleeding, Dissection–Coagulation Relay). Individual data points represent single task observations. SD, standard deviation

Both enrolled participants completed all study procedures; no participants were excluded or withdrew from the study. Mean total task time was 27.3 ± 10.9 min with Symphera compared to 33.9 ± 13.2 min with Control, representing a reduction of 6.7 min (19.6%). This pattern was consistent across both participants: Participant 1A completed tasks in 27.5 ± 10.7 min (Symphera) versus 34.4 ± 12.9 min (Control), and Participant 2B in 27.0 ± 12.1 min versus 33.4 ± 14.7 min respectively (Fig. 1). The Wilcoxon signed-rank test did not reach conventional significance (Hodges–Lehmann estimate −6.7 min, 95% CI −12.4 to −1.5 min; *p* = 0.10). The total number of tool changes was slightly higher with Symphera than with Control (27.1 ± 16.8 vs. 24.0 ± 14.1; + 13.0%; Hodges–Lehmann estimate + 2.9 changes, 95% CI + 0.3 to + 5.0; *p* = 0.10). (Table 1) Total tool change time was reduced with Symphera (3.6 ± 2.3 min) compared to Control (9.4 ± 6.6 min), a reduction of 61.5% (Hodges–Lehmann estimate −5.2 min, 95% CI −9.7 to −3.4 min; *p* = 0.10). This reduction was a data pattern consistent across both participants, and whilst it did not reach statistical significance, was most pronounced for Participant 2B, whose mean change time was 2.6 ± 1.5 min (Symphera) versus 10.1 ± 7.1 min (Control).

**Table 1:** Summary of procedural efficiency outcomes by condition

Variable	Condition	Mean ± SD	Median	*p*-value (Wilcoxon)
Number of tool changes	Control	24.0 ± 14.1	21.5	0.10
Number of tool changes	Symphera	27.1 ± 16.8	26.5	0.10
Total change time (min)	Control	9.4 ± 6.6	8.5	0.10
Total change time (min)	Symphera	3.6 ± 2.3	3.5	0.10
Total task time (min)	Control	33.9 ± 13.2	36.3	0.10
Total task time (min)	Symphera	27.3 ± 10.9	25.3	0.10

A task-level descriptive breakdown is presented in Table 2. Across all three tasks, mean change time per exchange was consistently lower with Symphera, ranging from 6.8 to 10.8 s compared to 20.2 to 23.5 s with Control. The total task time was reduced in the Symphera group across all three tasks but varied in magnitude: Task 1 (−7.2 min, 25.6%), Task 3 (−11.5 min, 24.3%), and Task 2 (−1.3 min, 5.0%). The number of extracorporeal changes was markedly lower with Symphera across all tasks, though the overall number of instrument exchanges was increased.

**Table 2:** Task- and participant-level breakdown of procedural efficiency outcomes (mean (SD))

		Task 1 (Cholecystectomy)		Task 2 (Hepatic bleeding)		Task 3 (Dissection–Coagulation)	
Part	Variable	Control	Symphera	Control	Symphera	Control	Symphera
1A	Mean change time (s)	22.0 (4.2)	13.0 (4.2)	24.5 (7.8)	14.0 (0.0)	23.0 (5.7)	9.0 (2.8)
1A	Mean extracorporeal time (s)	22.0 (4.2)	95.0 (111.7)	24.5 (7.8)	56.0 (15.6)	23.0 (5.7)	75.0 (24.0)
1A	N extracorporeal changes	5.0 (2.8)	3.0 (1.4)	20.5 (2.1)	5.5 (2.1)	40.5 (2.1)	2.5 (0.7)
1A	Number of tool changes	5.0 (2.8)	8.0 (5.7)	20.5 (2.1)	26.5 (2.1)	40.5 (2.1)	43.0 (1.4)
1A	Total change time (min)	2.0 (1.4)	1.8 (1.6)	8.5 (3.3)	6.2 (0.5)	15.8 (4.7)	6.2 (1.6)
1A	Total task time (min)	32.2 (11.0)	20.7 (0.5)	24.4 (13.3)	29.8 (5.4)	46.5 (5.6)	31.9 (20.0)
2B	Mean change time (s)	18.5 (4.9)	5.5 (0.7)	22.5 (10.6)	7.5 (0.7)	24.0 (9.9)	4.5 (0.7)
2B	Mean extracorporeal time (s)	18.5 (4.9)	20.0 (15.6)	22.5 (10.6)	78.0 (90.5)	24.0 (9.9)	6.0 (8.5)
2B	N extracorporeal changes	12.0 (5.7)	2.0 (0.0)	26.0 (7.1)	3.5 (3.5)	40.0 (0.0)	1.0 (1.4)
2B	Number of tool changes	12.0 (5.7)	8.5 (3.5)	26.0 (7.1)	26.0 (4.2)	40.0 (0.0)	50.5 (2.1)
2B	Total change time (min)	4.0 (2.8)	0.8 (0.5)	10.4 (7.4)	3.3 (1.0)	16.0 (6.5)	3.5 (0.7)
2B	Total task time (min)	24.0 (12.9)	21.0 (5.3)	28.1 (15.2)	20.1 (13.3)	48.3 (1.3)	40.0 (4.9)

**Fig. 2:**
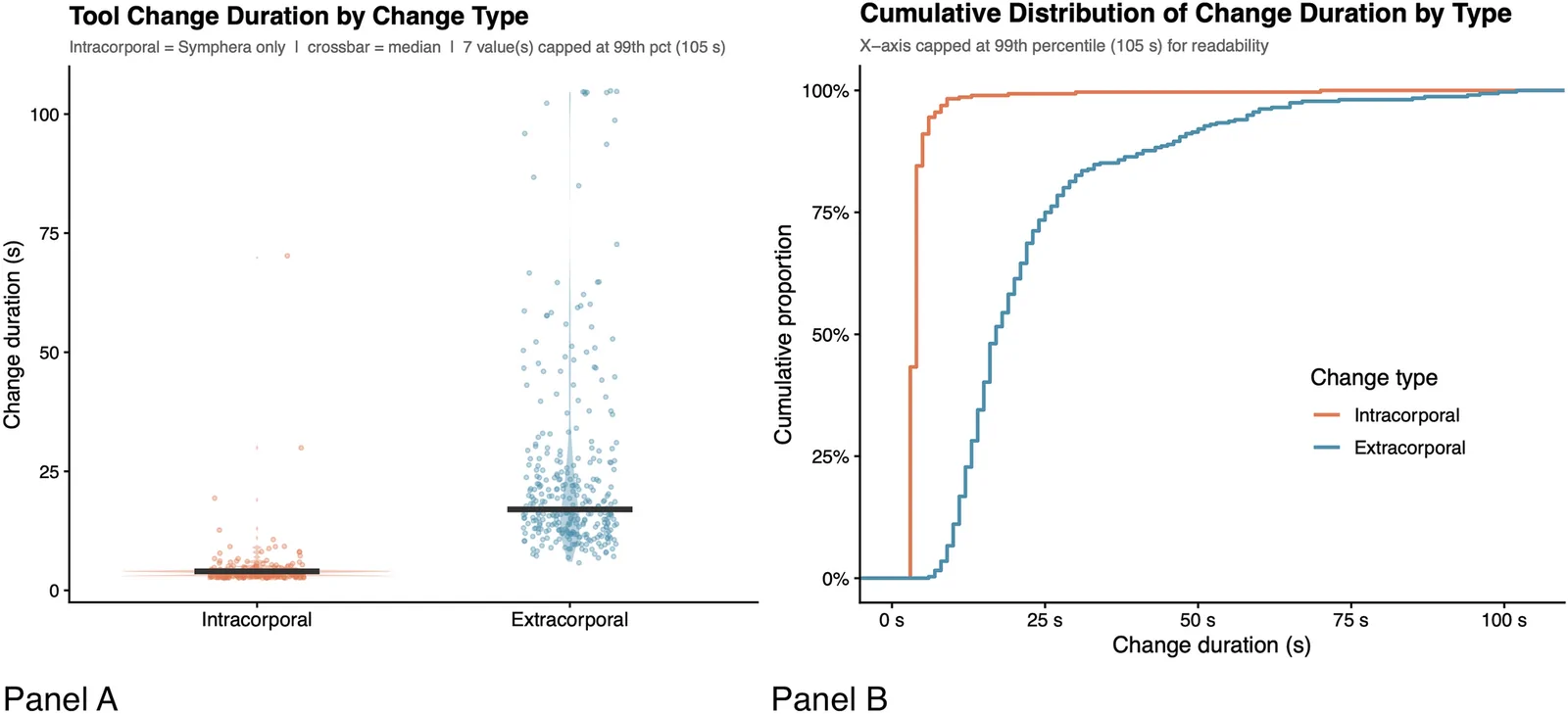
Distribution of individual tool change durations. Panel A: Mean tool change duration (seconds) for intracorporeal exchanges (Symphera condition) versus extracorporeal exchanges (pooled across both conditions). Panel B: Cumulative distribution of change durations by exchange type, illustrating that 75% of intracorporeal changes were completed within 5 s, compared to a broader, right-skewed distribution for extracorporeal exchanges. *SD*, standard deviation

A total of 614 individual tool change events were recorded across all procedures, comprising 291 intracorporeal changes (Symphera only) and 323 extracorporeal changes. Intracorporeal changes were substantially faster than extracorporeal changes, with a mean duration of 4.3 ± 4.4 s compared to 25.3 ± 23.4 s for extracorporeal changes (Fig. 2–Panel A).

The cumulative distribution of change durations confirmed this separation, with 75% of intracorporeal changes completed within 5 s, whilst extracorporeal changes showed a broader and right-skewed distribution extending up to 184 s (Fig. 2–Panel B).

When extracorporeal changes were compared between conditions, mean duration was longer in the Symphera condition (39.5 ± 31.0 s) than in Control (23.6 ± 21.8 s; Hodges–Lehmann estimate −8.0 s (Control shorter), 95% CI −16.0 to −3.0 s; *p* < 0.001).

**Fig. 3:**
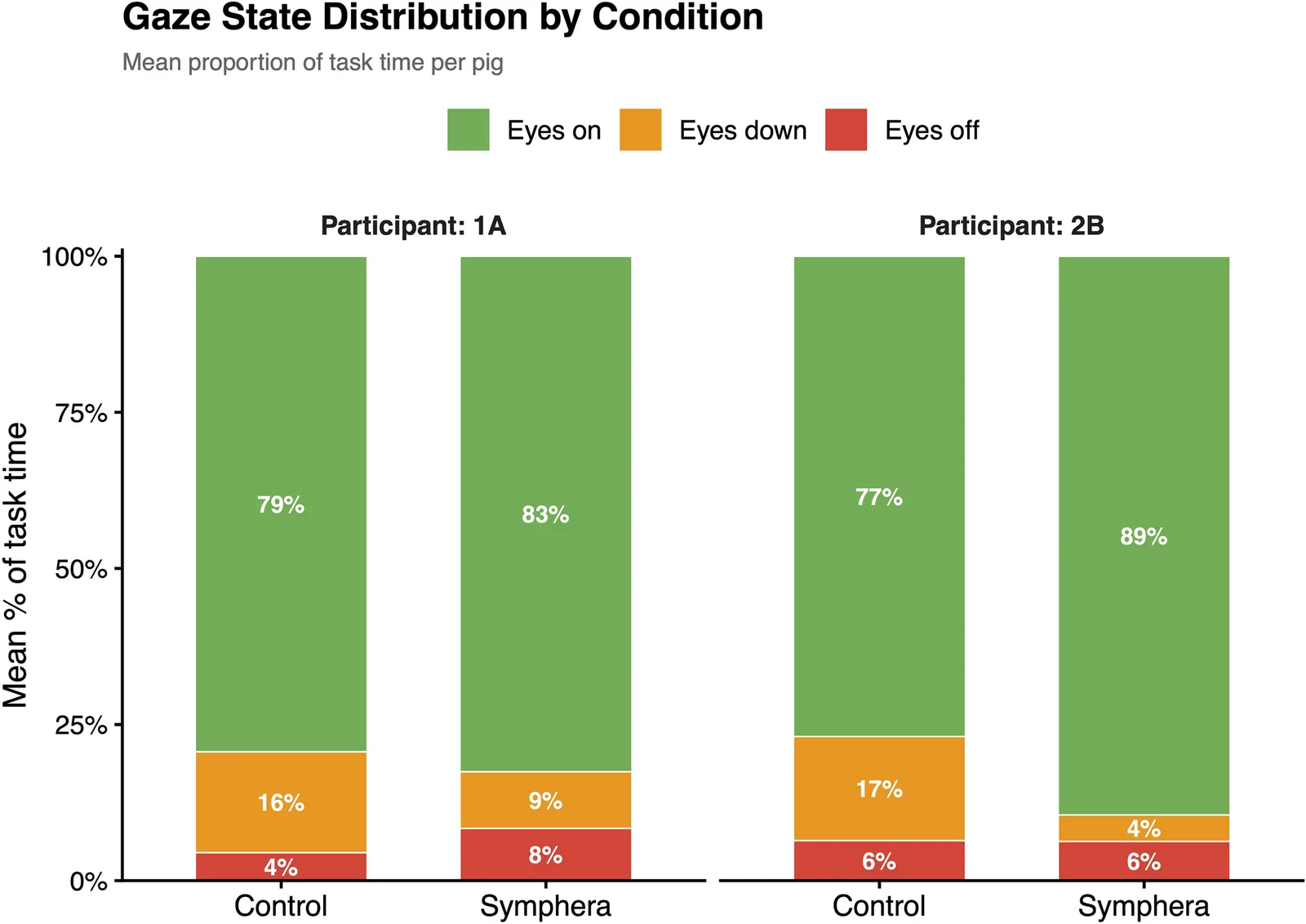
Eyes-on proportion (percentage of total task time with gaze directed at the laparoscopic monitor) by condition and participant. Higher values indicate greater sustained visual attention to the operative field. Individual data points represent single task observations per participant. Gaze classification followed the framework described by Ibbotson et al

Eyes-on proportion—the percentage of task time during which the surgeon’s gaze was directed at the monitor—was higher in the Symphera condition for both participants (Fig. 3). Across all procedures and tasks, mean eyes-on proportion was 86.0 ± 10.9% with Symphera compared to 78.1 ± 9.8% with Control. At the participant level, Participant 1A showed a mean of 82.6 ± 6.4% (Symphera) versus 79.4 ± 8.5% (Control), and Participant 2B 89.5 ± 13.8% versus 76.9 ± 11.7%. The Wilcoxon signed-rank test did not reach conventional significance for Participant 1A (Hodges–Lehmann estimate + 3.6 pp, 95% CI −3.5 to + 9.3 pp; *p* = 0.295) or for Participant 2B (Hodges–Lehmann estimate + 12.9 pp, 95% CI + 1.4 to + 23.7 pp; *p* = 0.093). A pooled Wilcoxon signed-rank test across all paired observations from both participants reached conventional significance (Hodges–Lehmann estimate + 7.7 pp, 95% CI + 0.9 to + 14.8 pp; *p* = 0.031).

The gaze state distribution confirmed this pattern: with Symphera, the proportion of task time classified as Eyes down (gaze directed down towards hands and device) was reduced relative to Control for both participants, from 16 to 9% for Participant 1A and from 17 to 4% for Participant 2B. The Eyes off proportion (gaze away from both monitor and hands) remained low and comparable between conditions across both participants.

Analysis of disruption reasons revealed a shift in the composition of gaze departures between conditions. In the Control condition, instrument exchange was the most frequent reason for leaving the monitor gaze state, accounting for 49% and 47% of recorded transitions for Participants 1A and 2B, respectively. In the Symphera condition, instrument exchange was markedly reduced in relative frequency (20 and 38%), whilst extracorporeal work became the most common disruption reason (59 and 52%), reflecting the smaller number of extracorporeal changes that did occur.

**Fig. 4:**
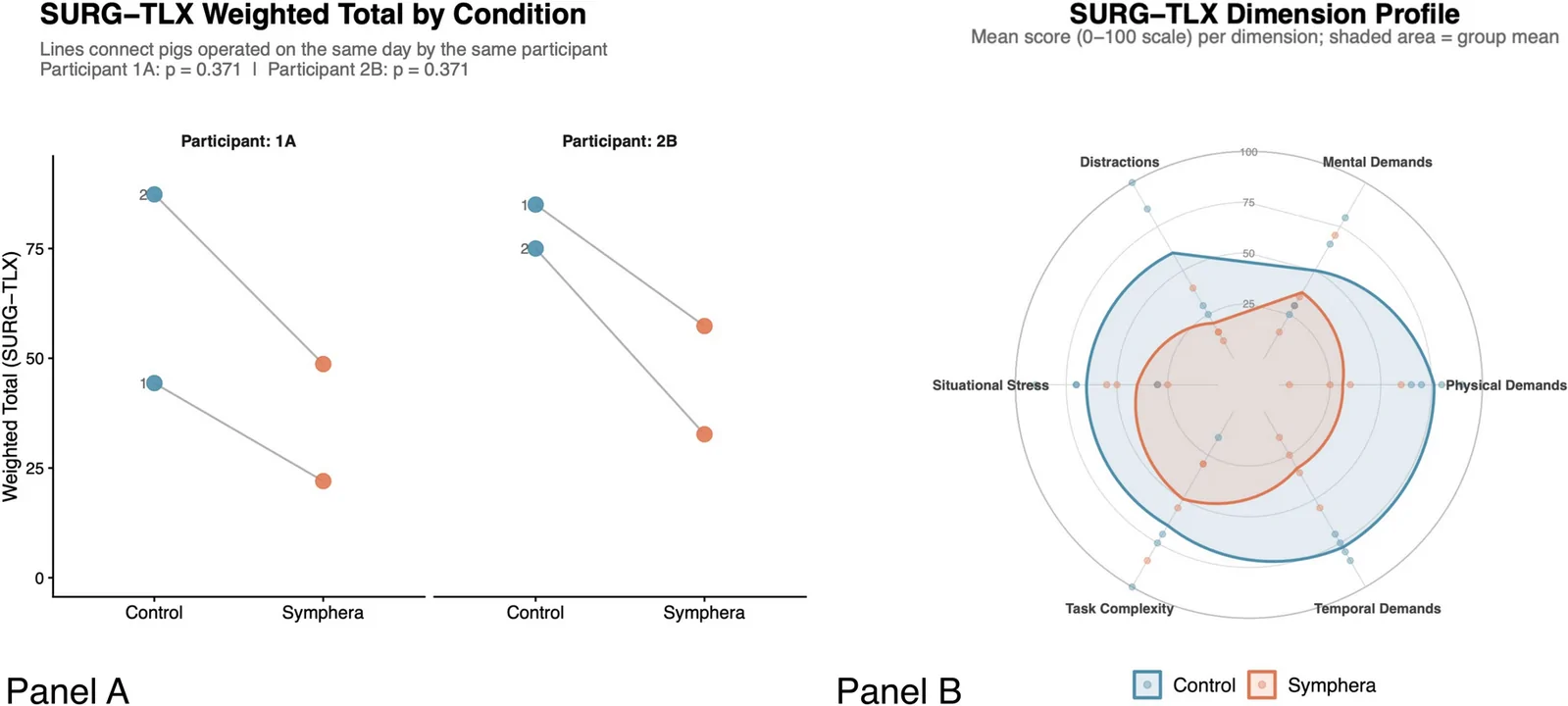
Subjective workload assessed by the Surgery Task Load Index (SURG-TLX). Panel A: Weighted total SURG-TLX scores (range 0–100) for each participant under Symphera and Control conditions; higher scores indicate greater perceived workload. Panel B: Dimension-level profile showing weighted scores across all six SURG-TLX dimensions (Mental Demands, Physical Demands, Temporal Demands, Task Complexity, Situational Stress, Distractions) by condition. SURG-TLX, Surgery Task Load Index

The SURG-TLX weighted total score was lower in the Symphera condition (40.2 ± 15.8; median: 40.7) compared to Control (72.9 ± 19.8; median: 80.0), representing a reduction of 32.7 points (44.8%; Hodges–Lehmann estimates: −30.5 points for Participant 1A and −35.0 points for Participant 2B; 95% CIs not estimable with *N* = 2 pairs; *p* = 0.371 for both). This pattern was consistent across both participants: Participant 1A reported scores of 35.3 ± 18.9 (Symphera) versus 65.8 ± 30.4 (Control), and Participant 2B reported 45.0 ± 17.4 versus 80.0 ± 7.1 respectively (Fig. 4–Panel A). Given the small sample, these findings should be interpreted as a strong exploratory signal rather than robust evidence of workload reduction.

The dimension-level profile revealed that the workload reduction with Symphera was present across all six SURG-TLX dimensions (Fig. 4–Panel B). Physical Demands, Temporal Demands, Situational Stress, and Distractions showed the highest absolute scores in the Control condition, whilst the largest reductions with Symphera were observed in these same dimensions.

Both participants completed the post-operative questionnaire following each operating day. Scores were reported on a direction-corrected 1–5 scale where higher values indicated a more favourable response.

**Fig. 5:**
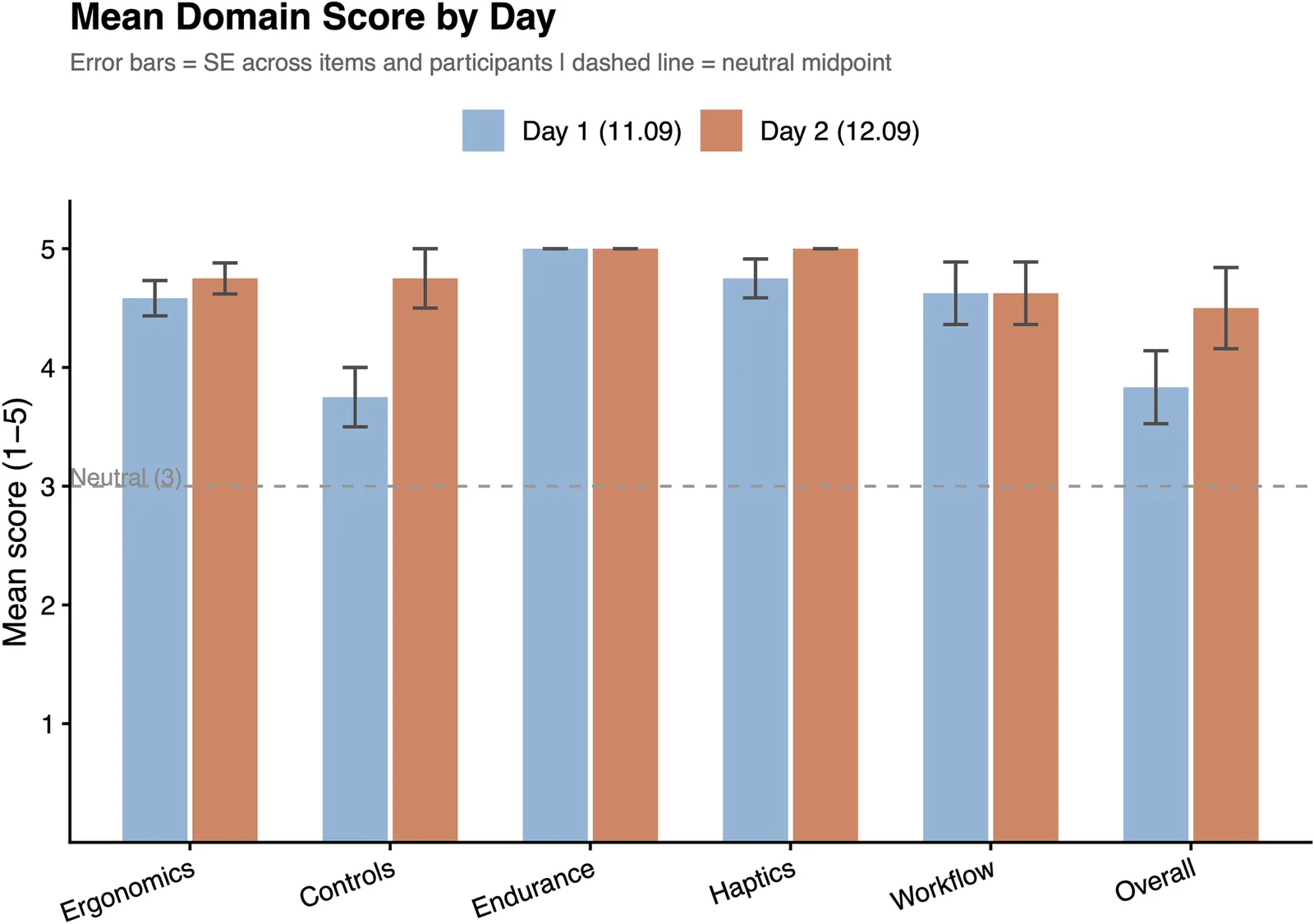
Post-operative device usability questionnaire scores by domain and study day. Scores are presented on a direction-corrected 1–5 scale, where higher values indicate more favourable ratings. Domains assessed: Ergonomics, Haptics, Endurance, Workflow, Controls, and Overall Assessment. Results from Day 1 and Day 2 are shown separately to illustrate changes across the study period

Domain-level scores were consistently above the neutral midpoint (3.0) on both days (Fig. 5). Ergonomics (Day 1: 4.58; Day 2: 4.75), Haptics (4.75; 5.00), Endurance (5.00; 5.00), and Workflow (4.63; 4.63) were rated favourably and remained stable across days. The Controls domain showed the largest improvement from Day 1 to Day 2 (3.75 to 4.75), driven primarily by control positioning (Q05: 3.0 to 5.0). The Overall Assessment domain improved from 3.83 to 4.50, with the overall device rating compared to standard instruments (Q17) rising from 4.0 to 4.5. The lowest-scoring item across both days was bipolar coagulation performance (Q19: 3.0 to 4.0).

**Fig. 6:**
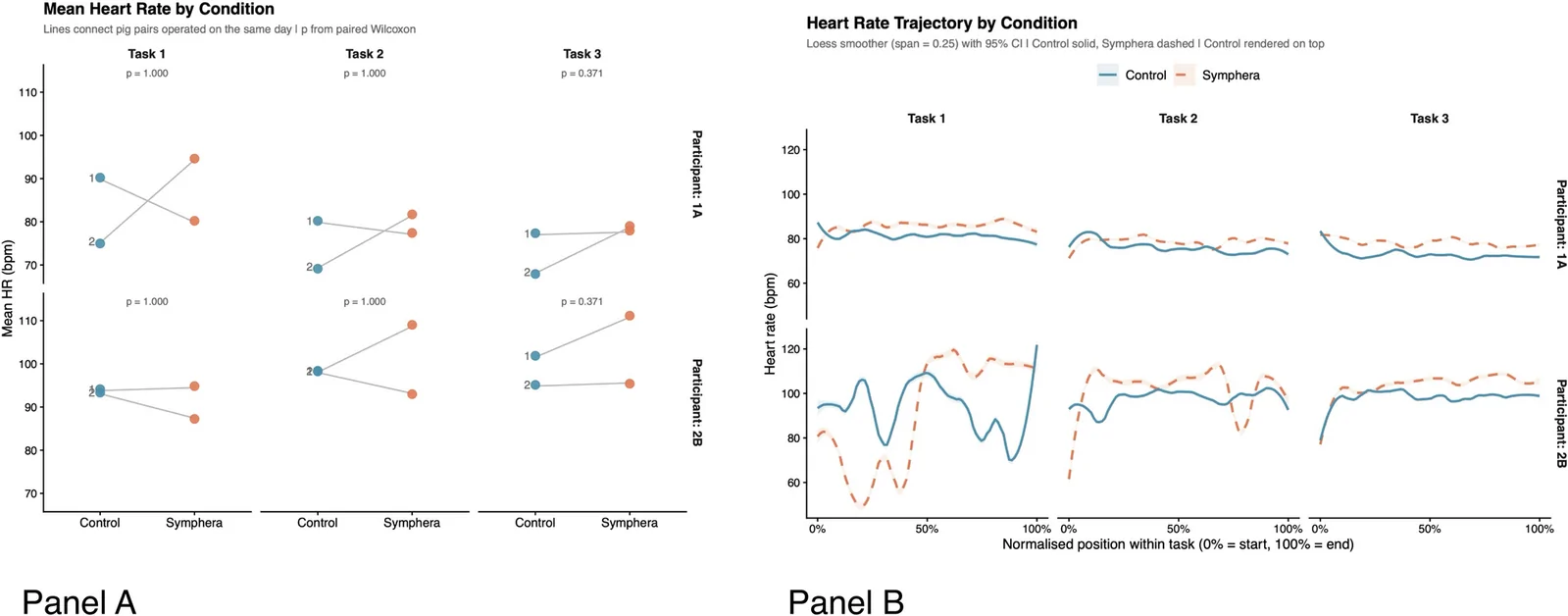
Surgeon heart rate (beats per minute) during task performance. Panel A: Mean heart rate by condition and participant across all procedures. Panel B: Within-task heart rate trajectories, with task duration normalised to a common time axis (0–100%) and mean heart rate computed at each normalised time point by condition. HR, heart rate; bpm, beats per minute

Mean heart rate was 89.9 ± 11.6 bpm in the Symphera condition and 86.6 ± 11.9 bpm in the Control condition across all procedures and tasks. At the participant level, Participant 1A showed a mean of 81.5 ± 6.5 bpm (Symphera) versus 76.5 ± 7.9 bpm (Control), and Participant 2B 98.4 ± 9.3 bpm versus 96.6 ± 3.2 bpm, respectively (Fig. 6–Panel A). The between-condition differences did not reach conventional significance for either participant (Hodges–Lehmann estimates: + 5.0 bpm for Participant 1A, + 1.8 bpm for Participant 2B; 95% CIs not estimable with *N* = 2 pairs; both *p* = 1.000). Within-task heart rate trajectories were broadly stable across the normalised task duration for both conditions and both participants (Fig. 6–Panel B). No systematic divergence between Symphera and Control was observed at any point within the task.

## Discussion

This exploratory study evaluated the Symphera prototype against conventional extracorporeal laparoscopic instrument exchange across three standardised laparoscopic tasks. Whilst most findings did not reach statistical significance—the pooled eyes-on analysis being the exception (*p* = 0.031)—there were discrete data patterns across procedural efficiency, gaze behaviour, subjective workload, and heart rate converging in favour of Symphera, lending preliminary support to the hypothesis that intracorporeal exchange reduces gaze disruption and perceived cognitive load whilst physiological arousal remains stable.

Mean intracorporeal change duration was 4.3 s compared to 25.3 s for extracorporeal changes—a nearly sixfold difference. The efficiency gain was most pronounced in the most exchange-intensive procedure (Task 3: − 24.3%) and in the cholecystectomy (Task 1: − 25.6%), whilst the structured repetition of Task 2 attenuated the benefit (−5.0%). Residual extracorporeal changes in the Symphera condition were on average longer than those in Control (39.5 ± 31.0 vs. 23.6 ± 21.8 s), attributable to the additional time required for instrument cleaning during these exchanges. Interestingly, the overall number of tool changes increased in the Symphera group, suggesting that surgeons may opt for a better-suited tool more readily when exchange carries less time cost. Whether surgeons conversely forego exchanges with conventional instruments to avoid the burden remains speculative and warrants investigation.

The Symphera barrel can be configured individually for each procedure, so the instrument set is not fixed. In the present study, the configuration was deliberately matched to the conventional comparator to ensure a fair crossover comparison; in clinical use, the configuration could be tailored per procedure—for example, by substituting a Maryland bipolar forceps for the Overholt dissector. A dedicated suction–irrigation tip is not currently available for the device but could represent a valuable future addition to further enhance operative efficiency and versatility.

Gaze analysis provided behavioural data consistent with the interpretation that fewer extracorporeal exchanges may be associated with preserved visual attention. Eyes-on proportion was higher under Symphera (86.0 ± 10.9% vs. 78.1 ± 9.8%), with Eyes-down time reduced from 16 to 9% for Participant 1A and from 17 to 4% for Participant 2B. The composition of gaze departures also shifted: instrument exchange, the leading disruption cause in Control (49% and 47% of transitions), fell markedly under Symphera (20% and 38%), replaced by extracorporeal work as the primary disruption category. This shift reflects the structural change that intracorporeal exchange introduces—collapsing the conventional withdraw–redirect–retrieve–re-engage sequence into a single in -situ operation that keeps the surgeon’s gaze anchored at the operative field, a pattern potentially relevant to situational awareness, though this requires investigation in adequately powered studies.

SURG-TLX weighted total scores were 44.8% lower under Symphera (40.2 ± 15.8 vs. 72.9 ± 19.8), with reductions present across all six dimensions and most pronounced in Physical Demands, Temporal Demands, and Distractions. This pattern is consistent with the hypothesis that fewer exchange-driven attention interruptions may reduce the accumulation of perceived cognitive load across an operative case. Device usability ratings were consistently favourable (all domain scores > 3.0 on Day 1, improving on Day 2), with the Controls domain showing the largest gain (3.75 → 4.75), a pattern that may reflect early familiarisation with the device. Bipolar coagulation performance was the lowest-rated item (3.0 → 4.0) and may warrant attention in subsequent prototype development.

Mean heart rate was numerically similar across conditions (Symphera: 89.9 ± 11.6 bpm; Control: 86.6 ± 11.9 bpm; *p* = 1.000), with stable within-task trajectories under both conditions. The small absolute difference (3.3 bpm) and the absence of HR peaks during Symphera use—despite participants operating an unfamiliar device—suggest that, in this small sample, the system was not associated with elevated physiological arousal, though the sensitivity of this measure is limited. Mean heart rate—in contrast to heart rate variability—is recognised as an insensitive index of cognitive load in surgical settings, being influenced primarily by physical effort and psychological stress rather than cognitive demand [23]. The stable and comparable HR values observed here therefore serve as a corroborating null signal: physiological arousal was maintained at a similar baseline whilst subjective and behavioural workload indicators showed directionally consistent differences in favour of Symphera. Additionally, the wrist-based optical monitors used here are subject to greater motion artefact and lower temporal resolution than chest-strap or ECG-based systems, further limiting the inferential value of these data.

The primary limitation is the small sample size (*n* = 2), which precludes statistical significance and generalisation; the study is explicitly hypothesis-generating. The study was not designed or powered to evaluate safety outcomes; no adverse events were observed, but safety conclusions cannot be drawn from two participants across 24 procedures. Both participants were IRCAD surgical fellows with no prior Symphera exposure, compressing the learning curve into the study period. The fixed task order introduces a potential sequence effect: as all three tasks were always performed in the same sequence, within-day adaptation, increasing familiarity with the porcine anatomy, and accumulating fatigue cannot be separated from the device effect. Performance improvements observed in later tasks may therefore partially reflect practice or familiarity rather than device benefit alone. The porcine model may additionally not fully capture the variability of live operative conditions. An additional limitation of this study is that the Symphera device in its current form does not allow for all types of laparoscopic instruments to be exchanged intracorporeally. Therefore, some specialised instruments, such as a needle driver or clip applier, would still require extracorporeal exchange, limiting the generalisability of these findings to all laparoscopic surgeries. Beyond the range of exchangeable instruments, further considerations intrinsic to a multifunctional device warrant acknowledgement as the technology matures. The device’s mechanical and software complexity may introduce failure modes and maintenance requirements not present with conventional instruments, and reliance on a single device means that a malfunction could simultaneously affect multiple functions. Acquisition cost may further affect adoption, and although reprocessing is simplified by a design in which only the reusable motor unit is sterilised whilst the remaining components are single-use, the consumable cost per procedure and the long-term durability and cost-effectiveness of the platform remain unproven. These considerations must be weighed against the potential benefits of fewer instrument exchanges, preserved visual attention, and improved workflow efficiency, and should be systematically evaluated in the future comparative and health-economic studies. A final limitation of this study design is that during the Control condition, the surgeon manually exchanged the instrument independently. In the operating room, a surgical assistant may be present to hand off the verbally selected instrument, which would likely decrease exchange time and gaze deviation.

## Conclusion

Across three measurement domains—procedural efficiency, gaze behaviour, and subjective workload—Symphera showed directionally consistent, favourable trends compared with conventional instrument exchange in this exploratory evaluation. The convergence of behavioural, subjective, and efficiency data is consistent with a mechanistic interpretation linking intracorporeal exchange to preserved visual attention and reduced perceived workload, and supports the rationale for larger confirmatory studies. Larger, adequately powered studies are warranted to evaluate whether these preliminary observations generalise to broader surgical populations and conditions.

## Supplementary Information

Below is the link to the electronic supplementary material.

Supplementary file1 (MP4 76595 KB)
